# Role of Macrodiols in the Synthesis and Thermo-Mechanical Behavior of Anti-Tack Water Borne Polyurethane Dispersions

**DOI:** 10.3390/polym14030572

**Published:** 2022-01-31

**Authors:** Nadia Akram, Muhammad Saeed, Muhammad Usman

**Affiliations:** Department of Chemistry, Government College University Faisalabad, Faisalabad 38000, Pakistan; msaeed@gcuf.edu.pk (M.S.); musman@gcuf.edu.pk (M.U.)

**Keywords:** polyurethane, anti tack, FTIR, DSC, DMA

## Abstract

The texture and molecular weight of polymer drastically affect the adhesion or tack strength. Waterborne polyurethane dispersions (WBPU) have been prepared using two different macrodiols of hydroxyl terminated polybutadiene (HTPB; Mn = 2912 g/mol^−1^) and four compositions of Polypropylene glycol (PPG Mn = 425, 1000, 2000, 2700 g/mol^−1^). The contents of the macrodiols have been varied using HTPB as 5, 10 and 15 mol%. The prepolymer of HTPB and Poly propylene glycol (PPG) have been developed using 4,4-Methylene bis(cyclohexyl isocyanate) (H_12_MDI) which is extended using 1, 4 butanediol (BD) followed by the dispersion of polymers in deionized water. Fourier Transform Infra-red spectroscopy (FTIR) is used to confirm the desired PU linkage. The probe tack graphs for tack analysis have not shown any plateau indicating absence of fibrillation. Two different values of glass transition temperature (*Tg)* have been observed for each dispersion using Differential Scanning Calorimetry(DSC). Storage modulus (E′) up to 3.97 MPa and (tanδ/E′) from 0.01–0.30 MPa^−1^ has been observed via Dynamic Mechanical Analysis (DMA). Introducing the HTPB has resulted in a decrease in the values of (tanδ/E′). No adhesion favorable parameters have been retrieved, indicating the molar variation a key factor in the development of anti-tack dispersions.

## 1. Introduction

Among a diverse range of viscoelastic materials, the water borne dispersions (WBDs) are vibrant facilitator in a number of applications; in particular, its usage is indispensable in the field of medicine. The unique operational properties of WBD allow it to be attached to the surface of a substrate with a slight pressure, which can even be applied with a finger, for a very short time, depending on the wettability of the adhesive and its potential substrate [1,2,3,4,5]. It is one of the abundantly used products in daily life [4,5,6,7]. Although WBD appears as a very common class of dispersions, its mechanism is actually more complex than anyone might perceive [5,6,7,8]. The unique wetting propensity assists to acquire obligatory contact with the substrate both on plane and bumpy surfaces alike contingent on the low modulus of elasticity. The optimum adhesion is complementary with a precise viscosity of adhesives enabling it to flow with ease on the top of the surface and to draw fibrils under high strain in limits. The meagre wetting is responsible for the stiffness of dispersions which develops short fibrils in contrast to adhesives. The fibrils produced from dispersions extend along the small strain values as the stress responsible to distort the fibrils is larger than the applied adhesive force on the adherent. Resultantly, it may lead to loss of tackiness. In cases of liquefied adhesive with the greater proportion of liquid, creep resistance will be lower under sheer stress. A good adhesive will lie in between these two extreme conditions. The ideal WBD requires a perfect balance of viscoelastic properties. The values of adhesion energy (*Ea)* emerge from dissipation of energy which is produced during the fibrillation process. It is also known as deformation process [6,9,10,11]. Among the various available tests for the measurement of tackiness, based on stress–strain curves, the probe tack test is significantly authentic as it provides a very clear picture of debonding process [7,12,13,14]. During the detachment of adhesive from the substrate a certain level of stress is required to resist the detachment purely dependent on the elastic behavior of the material. Surprisingly, both the viscous and elastic behavior are apparently opponents while a perfect WBD is an impeccable balance of both these properties [15,16,17].

Polyurethane is an excessively used polymer in a number of applications. The solvent borne PU dispersions are converted into water borne dispersions (WBD) without disturbing their colloidal stability and chemical nature. Certain factors, including reaction conditions, processing protocol, and especially the composition, alter the efficiency and the stability of the product. The solvent borne system can never be chosen over waterborne system due to environmental legislations. The eco-friendly nature and versatile capacity of PUD has made it a multipurpose product of industry [18,19]. The hydrophobicity of PU can be dealt easily by emulsification and by adopting some structural alterations [20,21,22]. The structural modifications can be performed by the macrodiols or diisocyanates. The diisocyanate should, however, maintain its stability in a moist environment to detect the hydroxyl of macrodiol instead of water or any other source to form urethane linkage by avoiding urea linkage [8]. Waterborne dispersions (WBDs) are not just restricted to adhesives; rather, these are replacing all the major solvent borne systems, including wood finish, top coatings on automobiles, and other industries, to minimize the consumption of solvents [23,24,25,26]. For every application, these materials need the right proportion of constituents.

Researchers have tried to understand the behavior of tack and anti-tack properties of various compositions when they are applied on the substrate and while they leave the surface of the substrate. Creton et al. [13] explored the detachment behavior of WBDs using probe tack experiments. The tack behavior was dependent on the *G*o/*E* parameter, indicating the importance of elastic modulus, and also elaborated the debonding mechanism through fibrillation and detachment. Crosby et al. [14] reported the adhesive behavior of elastic layers with lower thickness as compared to lateral dimensions. A thorough investigation anticipated three different deformation modes to understand anti-tack or non-adhesive behavior; (1) crack proliferation on edges, (2) interior crack proliferation, and (3) cavitation. The researchers have provided the details on each cracking mode to reveal the suitable conditions of stability. Yamaguchi and Doi [15] have established a three-dimensional mechanical model and determined the cavity extensions in a viscoelastic medium through the debonding process of the probe tack analysis.

While the researchers are discovering the debonding mechanism of WBDs, the novelty of this work is to quest for the factors which are silent contributors to reduce the efficiency of the adhesives by promoting anti-tack tendency and its impact on debonding. An entirely novel series of compositions has been developed to probe the question of tack strength. Our work highlights the need to explore the factor which actually hinders the efficiency of the product and, finally, decrease its efficiency. It is a very important issue to develop the right composition and to evade those contributors which will silently reduce the efficiency of the product and convert the dispersions into anti-tack products. Nevertheless, it is equally important to look at both aspects of any product, viz to develop a perfect consumer’s product and to minimize the factors reducing its efficiency. The goal of the work presented in this article is to evaluate the factors which may create a hindrance in the development of better adhesives. The elucidation of such factors will be helpful to open the new windows to develop a better product of WBD.

The research work presented here has been carried out by preparing waterborne polyurethane dispersions (WBPUDs). The samples were synthesized consuming two macrodiols of different chemical nature and molecular weight. One macrodiol of hydroxyl terminated polybutadiene (HTPB) consisted of hydrocarbon backbone. The M_n_ of this macrodiol is 2912 g/mol^−1^. The other macrodiol contains ether backbone chain known as PPG. The M_n_ of Poly propylene glycol (PPG) consumed for the development of the series is 425, 1000, 2000, and 2700 g/mol^−1^. The mole ratio of HTPB has been varied as 5, 10, and 15 mol% in combination with PPG of each molecular weight. This combination of macrodiols generated twelve different compositions while each was treated with 4,4-Methylene bis(cyclohexyl isocyanate) (H_12_MDI). The developed polymer samples were dispersed in deionized H_2_O. An extensive study of analyses was carried out to evaluate various factors responsible for anti-tack behavior of thin films of dispersions.

## 2. Materials and Methods

### 2.1. Materials

The following materials of analytical grade were used as received except macrodiols which were dehydrated in an oven at 80 °C prior to use; PPG (number-average molecular weight, M_n_ *=* 425, 1000, 2000, and 2700 g/mol^−1^ from (Sigma-Aldrich, St. Louis, MO, USA, B), HTPB (M_n_ = 2912 g/mol^−1^) BD (from Acros Organics, Geel, Belgium) [14]. H_12_MDI (Sigma-Aldrich, St. Louis, MO, USA), triethyl amine, TEA (Sigma-Aldrich, St. Louis, MO, USA), Dimethylol propionic acid, DMPA (Sigma-Aldrich, St. Louis, MO, USA), & Dibutyltin dilaurate DBTDL (Sigma-Aldrich, St. Louis, MO, USA.) [18].

### 2.2. Preparation of WBPUD

**Figure 1:**
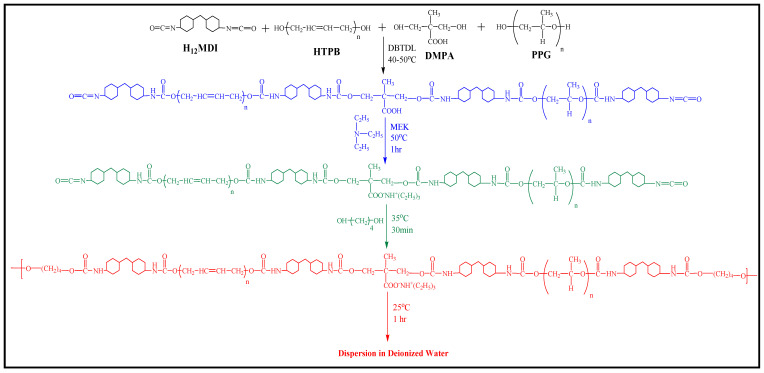
Synthetic scheme for the polymerization of water borne polyurethane dispersions (WBPDs) [21]. Black represents monomers; blue represents first stage of polymerization indicating formation of NCO terminated prepolymers. Green represents second stage neutralization of carboxylic group of DMPA with TEA; red indicates the fourth stage of chain termination with BD and dispersion in deionized H_2_O.

The prepolymer mixing methodology for the preparation of WBPUD was adopted as described in detail our previous publications [18,21,22,23]. A brief overview of the methodology is described; the WBPUDs preparation consisted of four steps. In the initial phase, both macrodiols (PPG and HTPB) were placed in a reaction vessel after degassing in a vacuum oven. The reaction vessel used for this particular reaction was a multi-neck round bottom flask connected with a high speed mechanical stirrer, a continuous nitrogen supply source, a thermometer, and a reflux condenser. The macrodiols, PPG and HTPB, diisocyanate H_12_MD, and DMPA were reacted according to stoichiometry reported in Table 1. The DBTDL was used as 1% of the total contents. The DMPA contains carboxylic group as shown in Figure 1 which was neutralized with TEA. As the DMPA has also provided the hydroxyl group, the functionality was utilized to formulate the composition of PUs. Hence, the functionality of DMPA was utilized in the formation of NCO terminated pre-polymer chains. The NCO terminated polymer chains were terminated by the use of BD as chain extender. A linear polymer chain structure was expected at this stage without any expected cyclic group formation due to the pre-selected NCO:OH ratio which has been selected in order to produce linear polymer chains. The prepared PU chains were dispersed in deionized H_2_O in order to complete the dispersion process in an aqueous medium. The detail scheme for the preparation of the samples is given in Figure 1. A series of WBPUD were prepared by following the same protocol while the stoichiometry of all the samples is given in Table 1. The architecture of PU consists of two parts; the soft segments (SS) consisted of macrodiols while the hard segments (HS) consisted of diisocyanates. In order to evaluate the adhesion tendency of dispersions the solid contents were maintained up to 40 wt.%. The sample codes, HS and SS composition is also reported in Table 1. All the synthesized samples were subjected to FTIR for structural analysis to evaluate the development of the right linkages. The samples were analysed by probe tack measurements in order to evaluate the adhesion capacity and to observe the debonding mechanism of the samples. The *Tg* of the samples were determined to evaluate its trend on the surface using DSC. The DMA studies were conducted to evaluate the mechanical behavior of the samples.

### 2.3. Characterization

#### 2.3.1. FTIR Analysis

FTIR measurements were carried out on a Nicolet 6700 spectrometer (Thermo Fisher Scientific Inc., Waltham, MA, USA) equipped with a deuterated triglycine sulfate detector (DTGS/KBr) equipped with Attenuated Total Reflectance (ATR) sampling accessory with a diamond crystal plate. Spectra were recorded in the spectral range of 4000–600 cm^−1^ at 4 cm^−1^ spectral resolution, spectral resolution, 2 sample gain, and 64 sample/background scans using OMNIC 8.1 computer software (Thermo Fischer Scientific Inc., Waltham, MA, USA).

#### 2.3.2. Probe Tack Adhesion Test

The test was performed as a reliable and quick test for tack evaluation. The WBPUD were pasted on glass strip (substrate) with 200 μm cube applicator and was dried for 24 h at room temperature. The thickness of the WBPUD films was sustained from 70 to 100 μm and was confirmed from digital calipers. The analysis was performed using TA-XT plus Texture Analyzer (Stable Microsystems, Godalming, UK). The test was performed using spherical stainless steel probe (diameter of 25 mm). During the test, the stainless steel probe touched the dispersion film for one second and was removed with a persistent velocity of 0.1 mm s^−1^ under a load of 4.9 N [19]. The optical microscope was used to determine the contact area on the substrate. By using the measures of the film thickness the work of adhesion *W_adh_* was calculated from the area under the curve [18,21,22,23].

#### 2.3.3. Differential Scanning Calorimetry

Differential scanning calorimetry (Q1000, TA Instruments, New Castle, DE, USA) was used in the range of −80 °C to 50 °C at a rate of 10 °C/min for the thermal analysis of samples. The analyses were carried out under nitrogen atmosphere. Heat flow and derivative heat flow curves were used to evaluate the data [18,21,22,23].

#### 2.3.4. Dynamic Mechanical Analysis

The films WBPUD of 2–3 mm in thickness were prepared in a Teflon mould and were dried in vacuum oven for one week. The analyses were carried out by DMA (Q800, TA Instruments, New Castle, DE, USA) in isothermal frequency sweep mode at 22 °C at a frequency of 1 Hz under 0.1 strain rate which was used for the measurements to evaluate storage modulus (E′), loss modulus (E″), and tan*δ* parameters [18,21,22,23].

## 3. Results and Discussion

### 3.1. Fourier Transform Infrared (FTIR) Spectroscopic Analysis

**Figure 2:**
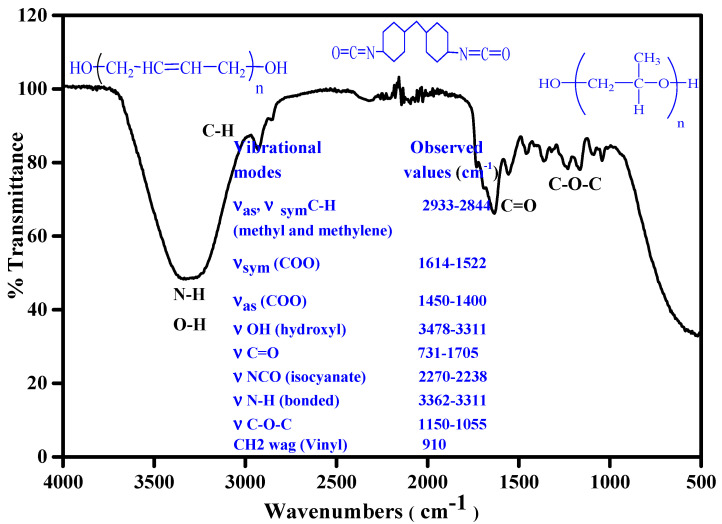
Representative FTIR spectrum of final stage of waterborne polyurethane dispersions (WBPUD) along with monomer structures and significant vibrational modes and corresponding wavenumbers (highlighted in blue).

The properties of the PU dispersions can be analysed from hydrogen boding which is developed due to inter and intra chain interactions. The hydrogen bonds are developed due the urethane linkage which contributes to the hydrogen bonding. The hydrogen bonding further dictates the size distribution and morphology of the PU chains. The increase in the hydrogen bonding results in better phase separation. The sensitivity of the FTIR in the carbonyl region provides a glimpse about the phase separation [27,28]. The formation of WBPUD takes place in multistep and it is responsible for the development of appropriate linkages. Hence, the analysis of FTIR technique is appropriate to form an idea about the development of anticipated linkages and can be considered as valid information about the confirmation of the desired product [18,24]. The FTIR spectrum of final stage for the formation of WBPUD is given in Figure 2, which represents the dispersion of PU chains in the deionized water. Figure 2 also shows that the developed linkages can be tracked from IR frequencies. The intensity of IR bands represents the amide and hydroxyl group. It also represents the absence of −NCO group, which is present due to DMPA. The presence of −OH functional group is observed in 3478–3311 cm^−1^, it is of prime interest that the OH group is present in the macrodiols, however the −OH of macrodiol reacts with −NCO of diisocyanate to develop urethane linkages generating −NCO terminated chains and these chains are later on terminated with −OH containing short chain diol. Moreover, the polymer chains are dispersed in water which has produced a broad band in the spectrum. The distinctive peak of methylene groups is observed at 2933–2844 cm^−1^ for both symmetric and asymmetric vibrations. The ether linkage C−O−C appears in the range of 1155–1055 m^−1^. The characteristic NCO peak of diisocyanate has been appeared at 2259–2256 cm^−1^ however, the vanishing of the IR peak shows the utilization of −NCO group into urethane linkage with the help of OH group. The characteristic carbonyl peak arises at 1731–1705 cm^−1^. The −NH group has shown multiple options to develop hydrogen bonding, which may be present either in “SS” or in “HS” segment contents. It is also noticeable that the hydrogen bonding of HS–HS is stronger as compared to HS–SS, making the HS interactions responsible for phase segregation [25,26].

### 3.2. Probe Tack Adhesion Analysis and Debonding Mechanism

**Figure 3:**
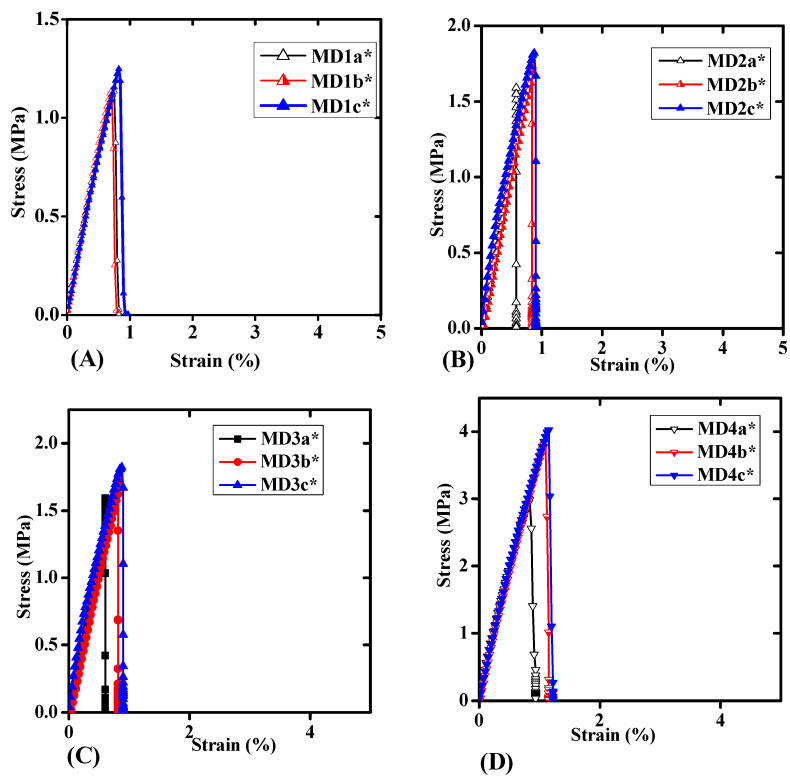
Illustrative probe tack curves of all the samples of MD Series of WBPUD. (**A**) shows representative probe tack curves of PPG Mn = 425 g/mol^−1^. (**B**) shows representative probe tack curves of PPG Mn = 1000 g/mol^−1^. (**C**) shows representative probe tack curves of PPG Mn = 2000 g/mol^−1^ (**D**) shows representative probe tack curves of PPG Mn = 27,000 g/mol^−1^. The letters with an asterisk (a*, b*, and c*) show concentrations of HTPB as 5 mol%, 10 mol%, and 15 mol%, respectively.

Figure 3 shows the representative curves of probe tack analysis for WBPUD. The test was conducted to analyse the adhesion/tack behavior of the samples. The figure represents the compositions of all samples with HTPB and PPG in different combinations. Figure 3 part A shows three representative curves of HTPB (Mn = 2912 g/mol^−1^) with PPG (Mn = 425 g/mol^−1^). For each concentration of HTPB; 5%, 10%, and 15 mol%, a linear trend was observed in the initial stage of stress–strain (σ-ε) curves until it attained the σ_max_ point of the curve. A sudden drop of σ_max_ curve was observed without any fibrillation. Surprisingly, the inception of cavities was observed for three samples of MD4a*, MD4b*, and MD4c* at 2.97, 3.87, and 4.06 MPa, respectively, which was highest among all the analysed samples. The similar trend of σ_max_ was observed regularly. However, neither of the curves produced a plateau, which confirmed the absence of fibrillation. The abrupt descent of the (σ-ε) curves indicated a quick crack development and its prompt propagation at low ε value. The values of ε were not significant. Apparently, no change in the representation of curve was observed. All the samples indicated a tack free behavior, suggesting a rigid and dried material with anti-tack properties. The samples have also shown the poor adhesion energy (*E_a_*) as the minimum *Ea* under the (σ-ε) curve was detected. The lower (*E_a_*) indicated that the material was non-stick in nature. It was also observed that the high molecular weight macrodiols have been resulted in anti-tack behavior [14,18].

The polarity of the monomers plays a significant role in the phase separation, the greater difference in the polarity of the components improves the chances of the phase separation. While considering the nature of the HTPB and PPG separately, it is important to note that HTPB (Mn = 2912 g/mol^−1^) contains an inert and nonpolar back bone in nature and show insufficient compatibility with extremely energetic monomers. The immiscibility of HTPB and insufficient interaction between the segments results in the lack of the connectivity of the fibrils or show the poor mechanical behavior, leading to breaking of the fibrils. The phase separation, in this case, can be observed by the introduction of crosslinkers at macroscopic and microscopic level. The crosslinkers acts as a bridge in between two monomers of different polarities. While there are several methods to improve the polarity of the HTPB using binders, introducing polyether can also improve the polarity. The main reason of the phase separation is the development of the urethane bonds. The high polarity of the urethane bonds promotes phase separation.

**Figure 4:**
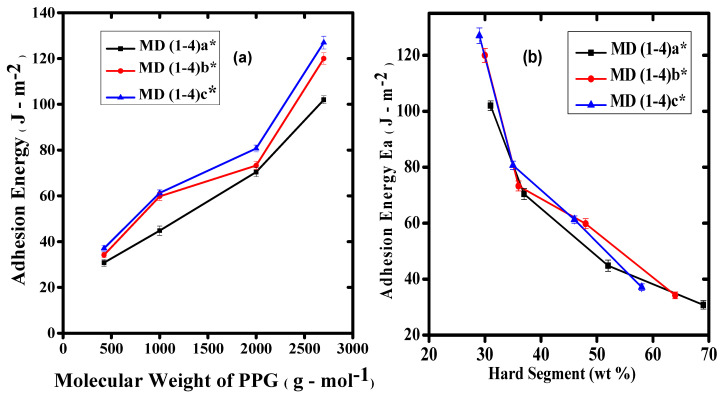
Graphical representation of parameter evaluated from probe tack curves MD Series of WBPUD; (**a**) dependence of *Ea* on molecular weight of PPG; (**b**) dependence of *Ea* on hard segment contents. Numbers 1–4 in both graphs show the molecular weight of PPG Mn = 425, 1000, 2000, 2700 g/mol^−1^, respectively. The letters with an asterisk (a*, b*, and c*) designate the concentrations of HTPB as 5 mol%, 10 mol%, and 15 mol%, respectively.

The relationship between “*E_a_*” vs. “M.wt” of PPG and “*E_a_*” vs. “HS” is given in Figure 4a,b. Figure 4a indicated a direct relationship between M.wt and *E_a_*, the *Ea* was calculated from area under (σ-ε) curves. An increasing trend in the *Ea* was observed with increase in the M.wt of PPG. The increased M.wt of the HTPB delayed the cavitation development resulting in a diminution in the fibrillation. The M.wt of HTPB is also responsible for entanglements of polymer chains. The upsurge in the concentration of HTPB helps to entangle the polymer chains of PPG resulting in restricted movements of the chain hindering the process. This type of behavior is also responsible for close chain packing and restricts the flow of polymer chains [12].

In case of Pus, the HS and SS contents have a great impact on the adhesive properties. The data are provided in Table 2. The HS contents were shared by H_12_MDI, BD, DMPA, and TEA while the SS content was contributed by both macrodiols PPG and HTPB. The higher M.wt of macrodiols have decreased the HS contents of PUs. The HS contents were varied from 29 to 69%. The highest values of HS contents (69%) were observed with lowest molecular weight of PPG *M_n_* = 425 g/mol^−1^. The lower proportion of HTPB 5% has also decreased the SS proportion. While the continuous rise in the concentration of HTPB has decreased the values up to 64% for 10 mol% of HTPB and then 58% for 15 mol% of HTPB. The HS ratio was decreased by the escalation in the M.wt of PPG. The lowest values of HS were obtained with the maximum increase in the M.wt of PPG *M_n_* = 2700 g/mol^−1^. Figure 3 exhibits the correlation between HS contents (wt.%) and the *Ea* for the samples. The graph showed an escalation in the *Ea* of the samples with a decrease in the HS contents. The continuous decrease in HS contents was due to intensification in the *Ea* of the samples. The sample MD1a* with the maximum HS contents of 69% showed the minimum *Ea* of 37.1 J/m^−2^ for this series. On the contrary, the sample MD4c* showed the minimum HS contents of 29% and the *Ea* for the system was 127 J/m^−2^. Rests of the samples were observed in between these upper and lower limits of *Ea* and HS contents. As the process was observed without fibrillation, high values of *Ea* just represent the strength of the material by the increase in the HS contents.

### 3.3. Thermal Stability of WBPUD Thin Films

**Figure 5:**
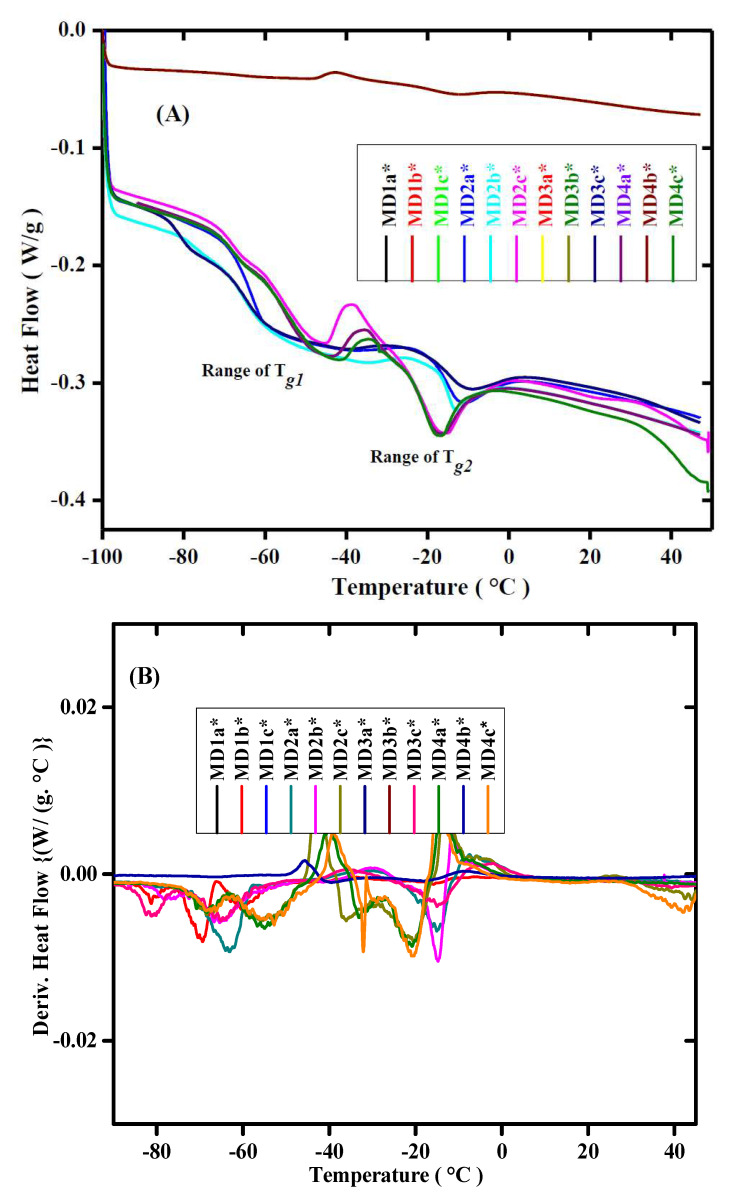
Thermograms of DSC of MD Series of WBPUD; (**A**) DSC thermograms of heat flow w.r.t temperature; (**B**) DSC thermograms of Derivative heat flow w.r.t temperature. Numbers 1–4 in both graphs shows the molecular weight of PPG Mn = 425, 1000, 2000, and 2700 g/mol^−1^, respectively. The letters with an asterisk (a*, b*, and c*) show concentrations of HTPB as 5 mol%, 10 mol%, and 15 mol%, respectively. Whereas, the asterisk symbol(*) represents the molecular weight of HTPB (2912 g/mol^−1^).

The thermograms of WBPUD from DSC have been shown in Figure 5. The thermograms predict the relationship between temperature and heat flow indicating at least two *Tg*. The presence of multiple Tg is associated with the presence of different monomers. The *Tg* values emerged from derivative heat flow curves from Figure 5B is given in Table 2. The composition of PU dispersions decides the major properties of the adhesives; the aliphatic diisocyanates are characterized with low *Tg* values as compared to higher *Tg* values of aromatic components. The molecular weight of the monomers significantly describes its behavior towards glass transition temperature, although under any circumstances the Tg is not completely dependent on molecular weight; various other factors, including the polarity of the monomers, presence of crosslinking, or hydrogen bonding, also influence the Tg of the polymers. The increase in the Tg drastically lowers the elasticity of the final product, which not only reduces the flexibility of the chains, but has a significant impact on the elasticity of the dispersions as well. The increase in the molecular weight of the macrodiols is responsible for the increase in the glass transition temperature of the final dispersions. The Tg of HTPB has been found as −70 °C, whereas the Tg of PPG has been recorded as −68 °C to −60 °C when Mn has been increased from 425 to 2700 g mol^−1^. As compared to monomers, an increase in the Tg values of PUD have been observed by increasing the molecular weight of the PPG as indicated in Table 2 from the values of MD1 to MD4. However, a slight decrease in the values of Tg has been observed in the WBPUD by increasing the quantity of HTPB (a* to c*). The increase in the molecular weight of macrodiols produces gel structures by developing crosslinking in the polymer network. These dispersions do not show significant thermal stability; however, the various components aid in the thermal stability of the thin films. One key factor is the contribution of HS and SS. In case of water borne dispersions, the miscibility of individual components, especially the macrodiols, can be compromised; the prime reason is the main back bone chain of the polymers is composed of non-polar hydrocarbon chains. However, the PU chains contain polar urethane linkages, hence the HS and SS predicts the phase separation of the PUs. The increase in SS is primarily responsible for the phase separation, hence the values in Table 1 suggest an increase in phase separation by the increase in the molecular weight of the PPG throughout in the series. This also strengthens the idea of block copolymers in the main chains of the polymers.

### 3.4. Mechanical Analysis and Relative Adhesion Trend (DMA)

**Figure 6:**
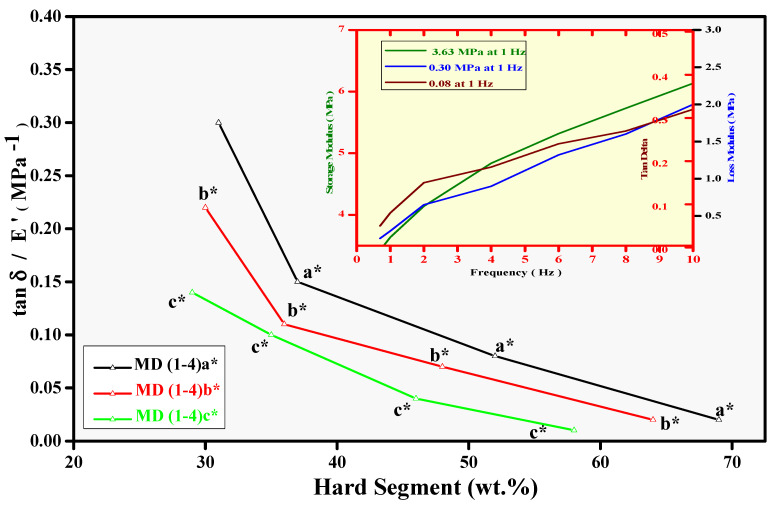
Correlation between Tanδ/E′ and HS contents of WBPUD, (Numbers 1–4 in both graphs show the M.wt of PPG Mn = 425, 1000, 2000, and 2700 g/mol^−1^, respectively; letters a, b, and c symbolise contribution of macrodiol HTPB as 5, 10, and 15, respectively, in moles. Whereas, the asterisk symbol(*) represents the molecular weight of HTPB (2912 g/mol^−1^) The inset shows original representative DMA curves of samples with E′, E″, and Tanδ w.r.t frequency.

**Figure 7:**
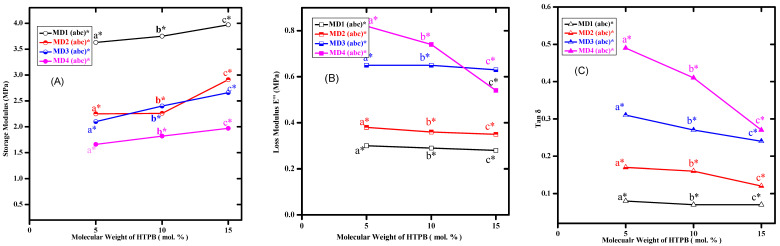
Representation of DMA parameters of MD series of WBPUD. (**A**) Relationship between storage modulus and molecular weight of PPG; (**B**) relationship between loss modulus and molecular weight of PPG; and (**C**) relationship between Tanδ and molecular weight of PPG. (Numbers 1–4 in both graphs show the molecular weight of PPG Mn = 425, 1000, 2000, and 2700 g/mol^−1^, respectively; letters a, b, and c symbolise contribution of macrodiol HTPB as 5, 10, and 15, respectively, in moles. Whereas, the asterisk symbol(*) represents the molecular weight of HTPB (2912 g/mol^−1^).

The dispersions are very soft material in nature. However, these are not devoid of mechanical features. The WBPUD films were analysed by DMA. Several important features related to mechanical strength have been observed, such as storage modulus (E′), loss modulus (E″), and damping factor (tanδ). As WBD is a very special type of adhesive, the adhesion is evaluated by a special parameter tanδ/E′ exalting the ability of adhesion of WBDs in connection with other parameters. Figure 6 shows the relationship between tanδ/E′ with HS contents. The values of all the samples of WBPUD have been extracted from original DMA graphs. A representative DMA graph has been shown in the inset of Figure 6. While the data of all the samples is given in Table 3. Figure 7A shows the relationship of E′ and M.wt of macrodiols, A decreasing trend in the values of E′ has been observed with M.wt of PPG. The values have indicated that the maximum value 3.97 MPa is actually far beyond the criteria of a good adhesive. Even the lowest value does not meet the standards of tackiness. Surprisingly, only 5% increment in the HTB molecular weight increase the E′ with increase in M.wt of PPG. All the adhesives lie in the range of 3.97 Mpa to 1.67 Mpa, indicating that no composition is actually showing adhesion. The greater E′ has indicated the rigidity in the film. Basically, the higher E′ has decreased the wettability of the adhesive layer, which in turn has produced lower *E_a_* and has reduced the tack ability of the adhesive. The higher E′ never favours the *E_a_*, and it produces a negative impact on the tackiness. The WBD, in a good condition, will have lower storage values and hence cannot be too rigid [16,18]. The higher values of E′ ultimately produce the crack and resultantly never initiate the growth of the cavities. The increase in the E″ values has been observed along with increase in M.wt of PPG in Figure 7B. A reduction in the E″ produces lower energy dissipation, which is the indication of absence of fibrillation process.

The relationship between molecular weight of macrodiols and tanδ is given in Figure 7C. It has endorsed the higher values of E″. However, the decrease in the concentration of tanδ has been observed with increasing concentration of HTPB resulting in decreased *E_a_*. None of the supportive factors could be extracted to promote tack or adhesion. Hence, the HS effect was neglected. However, the quantity of HS was not the only feature which has decided the better properties of the material; the nature of the monomers was equally important. Under the favourable conditions, tanδ/Eʹ improves the adhesive ability of the materials. As an outcome of high stiffness, the process of debonding is generally very slow, resulting in low tack ability.

## 4. Conclusions

The polymerization leading to the formation of good WBD is always a critical task which demands a lot of precautions to prepare the required material. Certainly, the right stoichiometry is very important in this connection. A mere change in the composition has a pronounced effect on the efficiency of final product. A series of WBPUD has been prepared with very sophisticated compositions, including two macrodiol of ether (PPG) and hydrocarbon (HTPB) nature, along with a cycloaliphatic diisocyanate H_12_MDI, using a prepolymer method of polymerization. The HS contents have been varied from 29 to 69 wt.%. A comprehensive experimental investigation has revealed the factors which can reduce the tack strength of the WBDs. The FTIR confirmed the correct sequence of linkage of urethane formation via characteristic carbonyl peak at 1731–1705 cm^−1^ and bonded amide at 3362–3311 cm^−1^. The adhesion and debonding mechanism revealed the non-adhesive behavior of the materials. The probe tack curves indicated that no cavities were developed in the adhesives; resultantly, no fibrils were produced, hence no tackiness was observed, though the *E_a_* was observed as 30.8–127 J/m^2^. It was a clear indication that adhesiveness was not entirely dependent on *E_a_*. This has confirmed that some of the ingredients in the composition were responsible for the non-tackiness of the materials. Most probably, it was the combination of high molecular weight macrodiols responsible for this non adhesive behavior. The DSC curves confirmed the presence of two *T_g_* in each sample; however, it was either too low or high to meet the criteria of good adhesive. The first glass transition temperature *T*_g1_ varied from −66 °C to −41 °C, while the second glass transition temperature *T_g_*_2_ varied from −19 °C to −6 °C. The DMA provided a very elaborative analysis of E′, E″, and Tan δ/E′. The graphs indicated higher storage modulus (3.97 MPa) as needed for a WBD. The Tan δ/E′ increased (0.02–0.30 MPa^−1^) with increase in the molecular weight of macrodiols. It was inferred that the molecular weight and composition of macrodiol was the primary factor for anti-tack behavior of WBD. It was coupled with a variation in *Tg* and mechanical properties which hindered the tackiness of the material, resulting in the deviation of conventional adhesion process.

## Figures and Tables

**Table 1:** Stoichiometry and segmentations of WBPUD.

Sample Code	Composition (Mole)				Hard and Soft Segment Contents (%)	
	PPG	HTPB	H_12_MDI	TEA	HS	SS
MD1 a,*	0.95	0.05	3	1	69	39
MD1 b,*	0.90	0.10	3	1	64	36
MD1 c,*	0.85	0.15	3	1	58	42
MD2 a,*	0.95	0.05	3	1	52	58
MD2 b,*	0.90	0.10	3	1	48	52
MD2 c,*	0.85	0.15	3	1	46	54
MD3 a,*	0.95	0.05	3	1	37	63
MD3 b,*	0.90	0.10	3	1	36	64
MD3 c,*	0.85	0.15	3	1	35	65
MD4 a,*	0.95	0.05	3	1	31	69
MD4 b,*	0.90	0.10	3	1	30	70
MD4 c,*	0.85	0.15	3	1	29	71

MD is devoted to the characteristic Diisocyanate (H_12_MDI, the digits shown with MD 1 to 4 embody the molecular weight of macrodiol PPG of *M_n_ =* 425, 1000, 2000, and 2700 g/mol^−1^, respectively; letters a, b, and c symbolise the contribution of macrodiol HTPB as 5, 10, and 15, respectively, in moles. Whereas, the asterisk symbol(*) represents the molecular weight of HTPB (2912 g/mol^−1^) HS symbolizes %HS = [(W_H12MDI_ + W_DMPA_+ W_TEA_+W_BD_)/W_Total_] × 100. %SS = 100-HS.

**Table 2:** Evaluation of adhesion parameters and glass transition temperature from Probe tack and DSC of WBPUD.

Sample Code	σ_max_ (MPa)	ε at σ_max_	ε _max_	W_adh_ (J/m^−2^)	*Tg*_1_ (°C)	*Tg*_2_ (°C)
MD1 a,*	1.14	0.69	0.75	30.8 ± 1.5	−65	−6
MD1 b,*	1.17	0.72	0.82	34.2 ± 1.2	−65	−14
MD1 c,*	1.26	0.81	0.93	37.1 ± 1.2	−66	−15
MD2 a,*	1.44	0.71	0.78	44.8 ± 2.0	−56	−20
MD2 b,*	1.63	0.81	0.97	59.8 ± 1.8	−63	−14
MD2 c,*	1.64	0.93	1.00	61.3 ± 1.3	−65	−10
MD3 a,*	1.61	0.59	0.60	70.4 ± 2.0	−43	−14
MD3 b,*	1.74	0.83	0.85	73.3 ± 1.8	−45	−16
MD3 c,*	1.82	0.87	0.94	80.7 ± 1.5	−45	−19
MD4 a,*	2.97	0.83	0.94	102.0 ± 1.7	−41	−20
MD4 b,*	3.82	1.10	1.12	120.0 ± 2.5	−43	−12
MD4 c,*	4.06	1.14	1.23	127.0 ± 2.8	−45	−18

MD is devoted to the characteristic Diisocyanate (H_12_MDI, the digits shown with MD 1 to 4 embody the molecular weight of macrodiol PPG of *M_n_ =* 425, 1000, 2000, and 2700 g/mol^−1^, respectively; the letters a, b, and c symbolise contribution of macrodiol HTPB as 5, 10, and 15, respectively, Whereas, the asterisk symbol(*) represents the molecular weight of HTPB (2912 g/mol^−1^) in moles. HS symbolizes %HS = [(W_H12MDI_ + W_DMPA_+ W_TEA_+W_BD_)/W_Total_] × 100. %SS = 100-HS.

**Table 3:** Viscoelastic parameters of WBPUD evaluated by DMA.

Sample Code	E′ (Mpa)	E″ (MPa)	Tanδ	tanδ/E′ (MPa^−1^)
MD1 a,*	3.63	0.30	0.08	0.02
MD1 b,*	3.75	0.29	0.07	0.02
MD1 c,*	3.97	0.28	0.07	0.01
MD2 a,*	2.25	0.38	0.17	0.08
MD2 b,*	2.26	0.36	0.16	0.07
MD2 c,*	2.91	0.35	0.12	0.04
MD3 a,*	2.10	0.65	0.31	0.15
MD3 b,*	2.40	0.65	0.27	0.11
MD3 c,*	2.66	0.63	0.24	0.10
MD4 a,*	1.66	0.82	0.49	0.30
MD4 b,*	1.82	0.74	0.41	0.22
MD4 c,*	1.97	0.54	0.27	0.14

Numbers 1–4 in both graphs shows the molecular weight of PPG Mn = 425, 1000, 2000, and 2700 g/mol^−1^, respectively. The letters with an asterisk (a*, b*, and c*) show concentrations of HTPB as 5 mol%, 10 mol%, and 15 mol%, respectively. Whereas, the asterisk symbol(*) represents the molecular weight of HTPB (2912 g/mol^−1^).

## Data Availability

Not applicable.
